# Climate Change and Older Americans: State of the Science

**DOI:** 10.1289/ehp.1205223

**Published:** 2012-10-02

**Authors:** Janet L. Gamble, Bradford J. Hurley, Peter A. Schultz, Wendy S. Jaglom, Nisha Krishnan, Melinda Harris

**Affiliations:** 1 U.S. Environmental Protection Agency, Washington, DC, USA; 2 ICF International, Washington, DC, USA

**Keywords:** adaptation, climate change, elderly, global warming, older adults, resilience, risk assessment, susceptible populations, vulnerability

## Abstract

Background: Older adults make up 13% of the U.S. population, but are projected to account for 20% by 2040. Coinciding with this demographic shift, the rate of climate change is accelerating, bringing rising temperatures; increased risk of floods, droughts, and wildfires; stronger tropical storms and hurricanes; rising sea levels; and other climate-related hazards. Older Americans are expected to be located in places that may be relatively more affected by climate change, including coastal zones and large metropolitan areas.

Objective: The objective of this review is to assess the vulnerability of older Americans to climate change and to identify opportunities for adaptation.

Methods: We performed an extensive literature survey and summarized key findings related to demographics; climate stressors relevant to older adults; factors contributing to exposure, sensitivity, and adaptive capacity; and adaptation strategies.

Discussion: A range of physiological and socioeconomic factors make older adults especially sensitive to and/or at risk for exposure to heat waves and other extreme weather events (e.g., hurricanes, floods, droughts), poor air quality, and infectious diseases. Climate change may increase the frequency or severity of these events.

Conclusions: Older Americans are likely to be especially vulnerable to stressors associated with climate change. Although a growing body of evidence reports the adverse effects of heat on the health of older adults, research gaps remain for other climate-related risks. We need additional study of the vulnerability of older adults and the interplay of vulnerability, resilience, and adaptive responses to projected climate stressors.

Older adults (i.e., individuals ≥ 65 years of age) are consistently identified as a population that is especially vulnerable to climate change stressors [Intergovernmental Panel on Climate Change (IPCC) 2007; U.S. Climate Change Science Program (CCSP) 2008; U.S. Global Change Research Program (GCRP) 2009]. A number of physiological, psychological, and socioeconomic factors contribute to this vulnerability, including the generally higher prevalence of certain diseases, medical conditions, and functional limitations among older adults; their higher sensitivity to extreme heat; their increased social isolation; and their financial status. The goal of this review is to summarize the current state of the science regarding the impacts of climate change on older adults in the United States, including key climate stressors; factors influencing the sensitivity, exposure, and adaptive capacity of older Americans to climate stressors; and measures that could be undertaken to reduce the vulnerability and enhance the resilience of older Americans to climate change. In this review, “sensitivity” refers to an individual’s or subpopulation’s responsiveness, primarily for biological reasons, to a given exposure. “Vulnerability” refers to the degree to which the ability of individuals or populations to cope with climate stressors is impaired. For our purposes, vulnerability is a function of sensitivity, exposure, and adaptive capacity.

## Methods

We performed literature searches using PubMed (http://www.ncbi.nlm.nih.gov/pubmed) and Google Scholar (http://scholar.google.com/) on demographics, relevant climate stressors, factors contributing to the vulnerability of older adults to those stressors, and response strategies. We included peer-reviewed papers and government reports dating from 2000 through 2011, but also included a number of earlier seminal articles. We developed three sets of search terms, and records returned were required to include at least one word from each of the three sets of terms. The first set describes the older adult population: aging, activity, daily living, assisted living, older adult, elder, old age, baby boomer, senior citizen, retirement community, nursing home, older population. The second set describes impacts, events, and potentially vulnerable areas: air quality, ambient pollution, coastal zone, coastal flood, drought, extreme event, extreme heat, extreme weather, flood, forest fire, global change, global environmental change, global warming, greenhouse gas, climate change, heat wave, heavy precipitation, high wind, hurricane, Katrina, natural disaster, ozone, particulate matter, sea level rise, storm surge, torrential rain, tropical storm, urban heat, wildfire. The third set describes effects on health and well-being: asthma, cardiovascular, chronic obstructive pulmonary disease, chronic illness, cognitive disorder, cold sensitivity, mental deficit, disability, disabled, displaced, displacement, evacuate, evacuee, heat sensitivity, heat stress, heat stroke, indoor mold, insect disease, Katrina cough, mental illness, post traumatic stress disorder, PTSD, relocate, respiratory.

We sought input from subject matter experts to review the overall search strategy and to locate additional citations. We also reviewed major synthesis reports, such as those by the IPCC, the GCRP, and the CCSP, to identify relevant citations. In total, we identified 430 publications. More than 300 citations were excluded because of an irrelevant title or because, on review, the abstract did not meet the inclusion criteria. We selected a more tractable set of 94 papers and reports that focused on and best reflected the topics outlined for this review, including demographic factors that influence vulnerability; physiological factors that affect sensitivity; climate stressors that affect older adults; factors that determine exposure to climate stressors; determinants of adaptive capacity; and decreasing vulnerability by improving resilience and adaptive capacity. We summarized key findings from identified papers and reports to prepare this state-of-the-science review.

## Results

### Demographic Factors that Influence the Vulnerability of Older Adults

Changes in climate during this century will be superimposed on a dramatic shift in the age distribution of Americans (with significant increases among the old and the very old), their geographic distribution, socioeconomic status, and other demographic changes.

*Who are older adults?* Older adults in the United States are a diverse group (Geller and Zenick 2005). Distinct subpopulations can be identified by age, race/ethnicity, socioeconomic status, degree of community and family support, general health, level of disability, and other characteristics (Federal Interagency Forum on Aging Related Statistics 2010; Pray et al. 2010). Older adults are expected to differ by birth cohorts, with today’s older adults and those of the future likely to vary in important ways. For example, the percentage of older Americans with at least a high school education is increasing even as the percentage of those living in poverty is decreasing. If these trends continue, they could lead to significant differences in relative vulnerability between the older adults of 2040 and the current elderly population (Pray et al. 2010).

**Figure 1:**
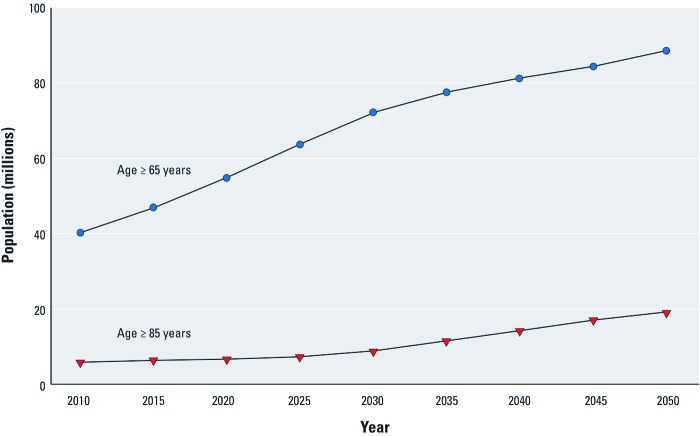
Projected population of older adults, 2010–2050. Data from U.S. Census Bureau (2008).

*America’s shifting age distribution.* The older adult population in the United States is increasing dramatically (Figure 1). In 2009, approximately 39.5 million Americans were ≥ 65 years of age (the old); the projected population of older Americans in 2050 is 88.5 million, 19 million of whom will be ≥ 85 years of age (the very old) (Pray et al. 2010). Older adults currently account for about 13% of America’s population, but are projected to account for 20% by 2040. The projected population shift is attributable in part to the aging of the baby boom generation, as well as increases in longevity and survivorship among older Americans, and is particularly apparent in the projected increase among the very old, those ≥ 85 years of age (Pray et al. 2010).

*Geographic distribution of older Americans.* Some locations with growing older adult populations are likely to be increasingly affected by climate stressors, such as hurricanes, droughts, floods, infectious disease, and rising summer temperatures. Zimmerman et al. (2007) reported that more than half of the older adult population was concentrated in 170 counties (5% of all counties) in 2005. Moreover, about 20% of older Americans resided in a county where a hurricane or tropical storm was likely to make landfall over the 10-year period from 1995 through 2005. There also appeared to be a higher concentration of low-income older adults in at-risk locations (Filiberto et al. 2009; Zimmerman et al. 2007).

In 2008, 51.2% of Americans ≥ 65 years of age lived in just nine states, with California, Florida, New York, Texas, and Pennsylvania accounting for the top five (U.S. Department of Health and Human Services 2009). These states could be especially hard hit by changing patterns of precipitation and storms. An increase in the severity of tropical storms and hurricanes could pose particular risks in Florida, where storms have historically resulted in significant property damage, injuries, and lives lost. In 2000, Florida had an older adult population of 16.8%, nearly 4% higher than the national average (Donner and Rodriguez 2008).

If current settlement patterns continue, future populations of older Americans will increase significantly in the Southwest and Southeast, along parts of the Atlantic and Gulf coasts, and in cities of the Northeast and Midwest that are expected to experience more frequent extreme heat events [U.S. Environmental Protection Agency (EPA) 2007]. To the extent that they are not physiologically or behaviorally adapted to high ambient temperatures, older Americans in northern cities may be particularly vulnerable to heat waves (Zanobetti and Schwartz 2008).

Other geographic factors influence the degree to which older adults are affected by climate change. Urban location is considered a risk factor for vulnerability to climate stressors, due in part to the exacerbation of summer heat by the urban heat island effect (a term that describes the relative warming of urban areas compared with their rural surroundings due to the replacement of open land and vegetation with buildings, roads, and other dark or impermeable surfaces) (CCSP 2008). Other geographic risk factors include urban sprawl (Stone et al. 2010), characteristics of the built environment, and perceptions of neighborhood safety (Browning et al. 2006).

### Physiological Factors that Influence the Sensitivity of Older Adults to Climate Stressors

Although many adults remain healthy and active long into their later years, old age is generally accompanied by an increased risk of certain diseases and disorders, along with changes in social factors such as increased social isolation and income loss. In this section we describe physical impairments that have been identified by multiple studies as risk factors affecting the sensitivity of older adults to climate stressors. “Sensitivity” refers to an individual’s or subpopulation’s responsiveness, primarily for biological reasons, to a given exposure. Although these risk factors are not confined to older adults, they may be more prevalent in the elderly as compared to the general population.

Some physiological limitations are functions of the normal aging process, but climate change may aggravate some impairments. For example, early-life exposure to air pollution (e.g., ground-level ozone) may increase the risk of respiratory and cardiovascular disorders with age (Filiberto et al. 2010; Wang et al. 2010). Similarly, early-life exposure to environmental toxicants whose concentrations may be affected by climate change (e.g., flood-related contaminants in drinking water) (CCSP 2008; IPCC 2007; Jardim 2011) may leave affected individuals with compromised immune systems that increase their sensitivity to climate stressors in later life. Aging itself is frequently accompanied by medical conditions that may aggravate susceptibility to infectious diseases (CCSP 2008; Wang et al. 2010).

*Respiratory impairments.* Because climate change can lead to increases in ground-level ozone and higher atmospheric concentrations of fine particulates, such as dust and allergenic pollen, in drought-prone areas individuals with respiratory impairments may be more at risk (CCSP 2008; IPCC 2007). Declines in respiratory function frequently accompany aging. Older adults may be more sensitive than the general population to adverse health effects from air pollutants and airborne pathogens and allergens (Filiberto et al. 2010; Wang et al. 2010). Even in the absence of disease, the lungs of older adults undergo physiological changes with age that can impair breathing (Wang et al. 2010).

Sensitivity to ozone varies with age and can affect lung function even in healthy older adults (Wang et al. 2010). Studies have documented associations between acute exposure to ambient ozone and increased risk of death, as well as an increased number of emergency room visits and hospital admissions among older adults (Arbex et al. 2009; Wang et al. 2010). Air pollution can exacerbate asthma and COPD, and exposure to ozone and other criteria pollutants can also increase risk of heart attack among people with diabetes, persons who are obese, and nonsmoking older adults (possibly because fine particles may penetrate the healthy lungs of nonsmokers more easily than those of smokers) (Baja et al. 2010).

*Impairments associated with diabetes.* In 2009, 19.9% of older Americans 65–74 years of age had diabetes, up from 9.1% in 1980 [Centers for Disease Control and Prevention (CDC) 2011]. Age-adjusted death rates for diabetes have increased among older Americans by 29% since 1981. One study projects that the prevalence of diabetes in America could double by 2050, due in part to the aging of the U.S. population (Boyle et al. 2010). Individuals with diabetes are at higher risk for heat-related morbidity and mortality than the general population (Federal Interagency Forum on Aging Related Statistics 2010; Westphal et al. 2010).

*Cardiovascular and thermoregulatory impairments and heat sensitivity.* In 2008, 31.9% of older Americans reported having heart disease, and 55.7% reported having hypertension (Federal Interagency Forum on Aging Related Statistics 2010). Cardiovascular impairment can make older adults more sensitive to health complications from warmer summers, heat waves, and air pollution. In addition, aging can impair the mechanisms that control body temperature. This effect may be exacerbated by certain illnesses (Worfolk 2000) and medications, such as some psychotropic drugs and cardiac medications, that can compromise thermoregulatory capacity (Allen and Segal-Gidan 2007; CCSP 2008; Ebi and Meehl 2007; Federal Interagency Forum on Aging Related Statistics 2010; Worfolk 2000).

### Climate Stressors and the Vulnerability of Older Adults

Older adults are among the most vulnerable in the general population to the direct impacts of weather-related natural disasters (Pekovic et al. 2007). Below, we summarize climate stressors that may disproportionately affect older adults. Some of these impacts will also affect the general population, but, where indicated, older adults are expected to be more vulnerable (i.e., more sensitive and/or more highly exposed and/or less able to adapt).

*Extreme heat.* Climate models project that extreme heat events will become more frequent and intense and of longer duration in the decades ahead (GCRP 2009), especially in the higher latitudes, affecting large metropolitan areas in the Northeast and Midwest where populations are less well adapted (Luber and McGeehin 2008). Extreme heat events are a major source of climate-related risk for older adults (Ebi and Meehl 2007; Gosling et al. 2009; Green et al. 2001; Medina-Ramón and Schwartz 2007; Ostro et al. 2009), with older Americans experiencing disproportionate risks of heat-related mortality (Ebi and Meehl 2007; Oudin Åström et al. 2011). Other health outcomes from extreme heat events include heat exhaustion, heat stroke, dehydration, acute renal failure and nephritis, exacerbation of cardiopulmonary diseases, and potential aggravation of side effects of some medications such as beta blockers used to control blood pressure, some psychotropic medications, and certain drugs used to treat chronic obstructive pulmonary disease (COPD) (Bargagli et al. 2009; CCSP 2008; Ebi and Meehl 2007; Knowlton et al. 2009; Luber and McGeehin 2008; Worfolk 2000).

Between 1979 and 2004, 5,279 heat-related deaths were reported in the United States, occurring disproportionately among older adults (Thacker et al. 2008). Heat stroke occurs at rates that are 12–23 times higher in persons ≥ 65 years of age compared with other age groups (Applegate et al. 1981; Caruso and Posey 1985). Because of a lack of a clear case definition and the multiple factors that contribute to heat-related mortality, heat-related deaths may be underreported (Ostro et al. 2009).

In the case of extreme temperature events, our focus is on extreme heat. But climate change may also decrease the frequency of extremely cold periods. Older Americans may benefit from warmer winters, both in terms of reduced heating costs as well as reductions in health and safety risks associated with cold weather (Kodra et al. 2011).

*Hurricanes, floods, droughts, and other extreme weather events.* Climate change is likely to contribute to an increase in hurricane intensity and precipitation, as well as increases in the frequency of other extreme weather events in the United States that may disproportionately affect older adults (CCSP 2008; IPCC 2011). Nearly 60% of the flooding-related fatalities following Hurricane Katrina were among persons ≥ 65 years of age (Jonkman et al. 2009). A rapid needs assessment of older adults in Florida found that Hurricane Charley, a category 4 storm that struck in 2004, aggravated preexisting, physician-diagnosed medical conditions in 24–32% of elderly households (CCSP 2008).

Apart from the obvious risks of direct physical injury or death, extreme weather events may lead to a range of secondary health impacts including those that affect the availability and safety of food and water; interruptions in communications, utilities, and health care services (CDC 2004; Kessler 2007); and increased risk of wildfires after drought (Cannon and DeGraff 2009). Hurricanes and other severe weather events may also lead to mental or emotional trauma before, during, and following the event. Analyses of pre- and postdisaster cognitive status showed decreases in working memory for middle-aged and older adults (Cherry et al. 2009). Nursing home residents and staff have been found to have mental health needs even 5 months after a hurricane (Laditka et al. 2008). Studies have found flood exposure to be related to health decrements in older adults, with the persistence and extent of adverse health effects positively related to flood intensity and duration (Phifer et al. 1988).

The enduring impacts of extreme events can be significant. For example, flooding can result in contamination of drinking-water supplies, increased incidence of indoor mold and associated respiratory illnesses, and long-term relocation and property loss among affected populations (Cannon and DeGraff 2009; Institute of Medicine 2011; Kessler 2007; Laditka et al. 2008). Extreme events can also compromise health care services and social support systems (Sanders et al. 2003).

Finally, the need to evacuate a region ahead of approaching severe weather can also pose health and safety risks for older adults. In particular, logistical issues can hamper the safe evacuation of long-term care facilities. In addition to the challenge of securing appropriate transportation, the evacuating facility must ensure that the receiving facility can manage patients’ needs. The demographics of the population to be evacuated helps determine the resources required (Bascetta 2006). Successfully moving individuals from nursing and assisted-living facilities to a sheltering facility requires the transfer of essential patient information and resources, including medical records, medications, and medical equipment (CDC 2004). This process was particularly problematic and poorly coordinated during the evacuation for Hurricane Katrina (Laditka et al. 2008). During Hurricane Rita in 2005, a bus evacuating elderly nursing home residents from Houston to Dallas was involved in an accident that killed 24 people (Falkenberg 2005).

*Climate impacts on air quality.* Climate change can affect air quality by increasing the formation of ground-level ozone and by leading to higher atmospheric concentrations of fine particulates, allergens, and dust in drought-prone areas. These effects, though not restricted to older adults, are typically more severe due to preexisting medical conditions.

Assuming precursor emissions are held constant, ozone concentrations are expected to increase in the United States as a result of climate change (CCSP 2008; Jackson et al. 2010). Higher levels of ground-level ozone can exacerbate cardiopulmonary illnesses (Park et al. 2005), especially asthma and COPD and premature mortality from these diseases (Laumbach 2010). Ozone injures lung tissue and promotes airway inflammation, and may have a stronger effect on respiratory disorders than on cardiovascular disease (Laumbach 2010; Moolgavkar 2000).

The changing climate has led to earlier onset of and increases in the seasonal production of allergenic pollen in middle and high latitudes in the Northern Hemisphere (Intergovernmental Panel on Climate Change 2007). Higher concentrations of atmospheric carbon dioxide may also increase pollen production in ragweed, prolong the pollen season, and increase some plant metabolites that affect allergenicity (CCSP 2008). Higher temperatures are associated with longer ragweed pollen seasons across a broad swath of midwestern states from Texas to the Canadian border (Ziska et al. 2011).

Climate change may also lead to increases in the amount of airborne dust being transported across the Atlantic Ocean from the Sahara Desert to the Caribbean Sea. Older adults living in areas where drought has increased in frequency and/or severity may also be at greater risk because exposure to dust can exacerbate existing respiratory illnesses (IPCC 2007).

*Impacts on infectious diseases.* Through its impacts on natural systems, climate change can facilitate the spread or emergence of vector-, water-, and food-borne diseases in areas where they had been limited or had not existed previously. At the same time, climate change may create conditions that are inhospitable for a vector or pathogen, causing related diseases to moderate or to disappear altogether (GCRP 2009).

The abundance and distribution of some infectious disease vectors (e.g., fleas, mosquitoes, and ticks) may be affected by climate change (CCSP 2008). For example, in North America an increase in the abundance and range of deer ticks and a loss of biodiversity among animal hosts important in the disease transmission chain, could alter the prevalence of Lyme disease (Chivian and Bernstein 2004; IPCC 2007; LoGiudice et al. 2003). If the public health infrastructure is maintained or improved, such diseases are unlikely to cause major epidemics in the United States. Nevertheless, to the extent that older adults are more likely to have compromised immune systems, vector-borne diseases may pose a greater risk (CCSP 2008).

The incidence of water- and food-borne illnesses can be affected by increased flooding (e.g., through contamination of food crops by flood waters and contamination of drinking water from combined sewer overflow and agricultural runoff) and other stressors associated with climate change (e.g., warmer temperatures, which may enhance bacterial growth) (CCSP 2008). Although the outcome of many gastrointestinal diseases is mild and self-limiting, these diseases can be severe and even fatal among vulnerable populations, including young children, those with compromised immune systems, and older adults. In a 1985 study, children 1–4 years of age and adults > 60 years each made up about 25% of hospitalizations involving gastroenteritis, but older adults represented 85% of the associated deaths (CCSP 2008).

### Factors Determining Exposure to Climate Stressors

Several factors affect the degree to which older Americans will be exposed to climate stressors; these include socioeconomic characteristics, housing characteristics, adequacy of neighborhood infrastructure, and availability of social services.

*Variations in socioeconomic factors in relation to climate threats.* Socioeconomic characteristics, such as income level, access to social and health services, and level of education, can influence the risk of exposure and the capacity to adapt. Older adults living in poverty or on fixed incomes may experience greater exposure to the effects of warmer summers and heat waves (e.g., due to lack of air conditioning or the reluctance to use it because of operating costs), along with other extreme weather events (e.g., due to substandard housing) (Drechsler et al. 2006). Higher mortality from heat waves has been associated with poverty and lack of a high school education (Browning et al. 2006; CCSP 2008; Cutter et al. 2003). Failing to complete high school is a proxy both for lower income and literacy rates, because the latter may predict the success of risk communication. The median income of older Americans in 2008 was $29,744, compared with $56,791 for Americans < 65 years of age (U.S. Census Bureau 2009). Almost 3.7 million older Americans (9.7% of the older adult population) were below the official poverty level in 2008. Another 2.4 million (6.3%) were classified as “near-poor” (income between the poverty level and 125% of poverty level) (U.S. Department of Health and Human Services 2009). A supplemental poverty measure developed by the Census Bureau in 2011 shows a greater percentage of older Americans in poverty (12.7%) compared with the official 2010 measure (U.S. Census Bureau 2011b). Data on long-term trends, based on this 2011 measure, are not yet available.

A larger proportion of older women, compared with older men, live below the poverty level in the United States. In 2010, 10.7% of American women ≥ 65 years of age lived in poverty, compared with 6.7% of men of that age (U.S. Census Bureau 2011a). The disparity is even greater among Americans ≥ 75 years of age: 12.1% of women in that age group were living in poverty in 2010, compared with 7.5% of men (U.S. Census Bureau 2011a).

*Local or housing-related exposure considerations.* Depending on its condition, quality of construction, and amenities, housing can increase or decrease exposure and reduce or exacerbate the occupants’ risk of injury and illness from climate-related impacts. Manufactured or mobile homes are especially vulnerable to high winds and other storm damage (Cutter and Emrich 2006). Lack of access to air conditioning and homes with fewer rooms are significant risk factors for heat-related mortality. Often, older adults on fixed incomes can ill afford the costs of cooling their homes (Balbus and Malina 2009; CCSP 2008). In deteriorating urban neighborhoods, where safety and crime are at issue, some older residents may live in a state of “self-imposed house arrest” (Browning et al. 2006) that may prevent them from seeking needed help in their communities.

*Adequacy of infrastructure.* The condition of infrastructure, including utilities (e.g., telephone, gas, and electricity) and transportation, plays a vital role in determining exposure of older adults. Dramatic increases in electricity demand during heat waves can lead to brownouts or blackouts, as experienced during the Chicago, Illinois, heat wave in 1995 (Changnon et al. 1996). Interruptions in power can affect even those who are financially able to cool their homes. In addition, a lack of access to transportation can hinder evacuation during extreme weather events. Many older adults rely on public transportation. The existence, availability, adequacy, and resilience of public transportation systems will help to determine exposure (Haq et al. 2008).

*Availability of social services.* The availability of social services can affect older Americans’ exposure to climate stressors. Social services originate from multiple sources, including local community organizations, neighborhood associations, aid distribution centers, assisted-living facilities, hospitals, home-care providers, and government agencies. A lack of community-based financial resources can affect the quality and provision of services (Browning et al. 2006). Without adequate support, existing social services may be overwhelmed by the needs of older adults—as in the 1995 Chicago heat wave, when too few ambulances and emergency personnel were available to meet demands (Changnon et al. 1996).

### Determinants of Older Adults’ Adaptive Capacity

In this section we describe some of the key physiological and social factors, identified across multiple studies, that affect the degree to which older Americans—and the communities and services they depend on—can adapt to climate stressors. Adaptive capacity, together with sensitivity and exposure, collectively determine vulnerability.

*Functional limitations and mobility impairments.* Functional limitations—such as a decline in muscle strength, coordination, or cognitive function due to illness, chronic disease, or injury—may reduce older adults’ ability to respond effectively to climate stressors. In 2007, 42% of people ≥ 65 years of age reported one or more functional limitations (Federal Interagency Forum on Aging Related Statistics 2010). In contrast, about 19% of the general population in 2005 reported a functional limitation (Brault 2008). The prevalence of functional limitations varies by sex and age. Older women experience a higher rate of functional limitation (47%) than do older men (35%) (Federal Interagency Forum on Aging Related Statistics 2010). The disparity in rates has been attributed to the fact that women tend to live longer than men, have higher rates of co-morbidity, and have higher rates of chronic health conditions such as arthritis, depression, and Alzheimer’s disease (Newman and Brach 2001). Dementia in the United States increases from an estimated 5% of the population at 71–79 years of age to 37% at ≥ 90 years. Other cognitive impairments increase from 16% at 71–79 years of age to 39% at ≥ 90 years (Agarwal et al. 2009).

Many older adults suffer from impaired balance and decreased motor strength (Fernandez et al. 2002). Osteoporosis, a condition of compromised bone strength that increases the risk of fracture in affected individuals, is highly associated with aging (Pietschmann et al. 2009). In an extreme weather event, wildfire, or flood, it may be more difficult for victims with mobility impairments to respond, to evacuate (if necessary), and to recover (Fernandez et al. 2002). The risk of injury or death during or after evacuation is highest among older adults (Uscher-Pines et al. 2009).

*Economic status.* Another determinant of adaptive capacity is the older adult’s ability to financially support the implementation of response measures. Poverty is a primary contributor to social vulnerability (Cutter et al. 2003). Older adults can also be more vulnerable to property damage or loss due to lack of insurance, limited personal finances, and poor credit-worthiness (Fernandez et al. 2002). Older adults may lack the financial resources to prepare for or respond to climate-related risks (Browning et al. 2006). Similarly, the economic vitality of a community may determine its ability to support effective hazard mitigation and disaster recovery. In sum, economically disadvantaged seniors living in disadvantaged communities have fewer resources available to support adaptation (Cutter et al. 2003).

Racial and ethnic disparities contribute to the susceptibility of older Americans to climate change (O’Neill et al. 2005). A disproportionate percentage of some minorities live in poverty (Drechsler et al. 2006). A study of heat-related vulnerability in 50 U.S. cities identified several subpopulations as especially vulnerable to extreme heat, including African Americans, people with diabetes, and economically disadvantaged individuals (Medina-Ramón and Schwartz 2007). Older adults in these subpopulations have been linked to poorer health status in general, limited access to health care, and poorer housing conditions (Balbus and Malina 2009).

Because many older adults live on fixed incomes, they may be especially affected by increases in costs for energy, food, and out-of-pocket medical expenses—all of which may be aggravated by climate change. Food accounts for about 12–13% of older Americans’ annual household expenditures (Federal Interagency Forum on Aging Related Statistics 2010). Since 1985, about one-third of older Americans reported expenditures on housing and utilities that exceeded 30% of household income (Federal Interagency Forum on Aging Related Statistics 2010). Out-of-pocket expenses for medical care among older Americans increased by 61% from 1999 to 2009; health care accounted for 12.9% of total expenditures among older Americans in 2009, compared with 6.4% for all consumers (U.S. Department of Health and Human Services 2011).

*Living situation.* A person’s living situation has an impact on his or her adaptive capacity. With the rise of managed care, many older Americans with impairments, who previously might have been institutionalized, are now cared for at home. During or following a disaster, it may be difficult for first responders to identify, locate, and reach these dispersed populations (Fernandez et al. 2002).

Social isolation may affect the adaptive capacity of older adults to certain climate stressors (Bascetta 2006; Fernandez et al. 2002; Zimmerman et al. 2007). Isolation has been identified as a key risk factor for death during extreme heat events (Health Canada 2011). Older adults living in isolation, especially those with cognitive impairments or mental illnesses, may not receive emergency information or may underestimate the severity or urgency of warnings. One post–Hurricane Katrina study of older adults found that although > 75% of the study group listened to news and evacuation advisories before the landfall of Hurricane Katrina, one-third of them said they might not or definitely would not evacuate (Rosenkoetter et al. 2007).

Older Americans living alone may also be at higher risk for abuse from frauds or scams relating to home improvements or repairs before or after extreme weather events. They are also more likely to be disadvantaged in terms of their ability to evacuate because of a disability, limited income, or lack of transportation (Bascetta 2006; Zimmerman et al. 2007). About 30.5% of all noninstitutionalized older Americans in 2008 lived alone; 50% of women ≥ 75 years of age lived alone (U.S. Department of Health and Human Services 2009). In contrast, about 26% of the general U.S. population lives alone (U.S. Census Bureau 2007). Although 93.5% of older Americans live in the community, only 4.5% live in nursing homes and 2% live in assisted living facilities (Pray et al. 2010).

*Technology.* Communication tools and technologies, especially access to the Internet and social media, are important for transmitting key information before, during, and after extreme weather events. The availability and effectiveness of these technologies, as well as technologies and systems for rapid identification of at-risk older adults, can contribute to adaptive capacity. In situations where climate-induced pollution or climate-exacerbated diseases could affect older adults, the status, availability, and cost of preventive technologies may influence adaptive capacity (IPCC 2007).

*Infrastructure.* The nature and age of housing stock can affect adaptive capacity, as can other characteristics of the built environment such as urbanization, economic vitality, and development (Cutter et al. 2003). Decaying commercial infrastructure and overall community decline were positively correlated with higher levels of heat-related mortality in the 1995 Chicago heat wave (Browning et al. 2006). For frail members of the older adult population who depend on life support systems, such as oxygen generators, ventilators, or electric wheelchairs, reliable electricity generation and transmission systems are crucial. In multistory residential buildings, loss of electricity disrupts the operation of elevators, which makes it more difficult, if not impossible, for older adults to obtain food, medications, and other supportive services (Frumkin 2002). Power outages can also affect the availability of telephone service, pumped water in high-rise buildings, and public transit (Klinenberg 2003; Lin et al. 2011).

*Human and social capital and adaptive behaviors.* An active and diverse community contributes to its members’ well-being. During the Chicago heat wave, older residents may have felt more at ease accessing services in neighborhoods that were more socially and commercially vibrant (Browning et al. 2006). Areas with larger concentrations of older adults in the population tend to have lower mortality rates, possibly due to the benefits of a shared social support network (Browning et al. 2006). Race and ethnicity may determine social vulnerability through the lack of access to economic and social support resources, cultural differences, and marginalization of minority communities. In their Social Vulnerability Index, Cutter et al. (2003) specifically identify African-American race, in addition to old age, as indicators of reduced adaptive capacity.

*Interacting effects.* Factors affecting adaptive capacity rarely act alone. Multiple factors interact to determine the adaptive capacity of older adults to climate stressors. For example, low socioeconomic status and lack of knowledge of or access to supportive institutions can combine to effectively prevent older Americans from navigating complex procedures required to obtain assistance (e.g., financial aid and social services) following a natural disaster. This situation may be more common among widows or widowers who live alone and have limited experience in obtaining assistance because such tasks had previously been managed by their spouse (Dyer et al. 2008). Adaptive capacity depends not only on available resources, but also on effective risk communication.

### Reducing Vulnerability through Effective Adaptation

In this section we identify some of the adaptation measures that can be put in place to decrease older Americans’ vulnerability to climate extremes. These measures are based partly on prior experiences in responding to extreme weather events and disease outbreaks. More research is needed to document the relative effectiveness of adaptation measures and to identify and disseminate best practices.

*Information and education.* To successfully direct resources toward vulnerable individuals, registries of older adults and technological tools (such as geographic information systems) for quickly identifying them may be useful (Eisenman et al. 2007). Key indicators of vulnerability and preparedness may also be combined with surveillance data on older adult populations to inform prevention and preparedness strategies (English et al. 2009). While authorities and supporting organizations need to know the location and condition of older adults, individuals need access to the knowledge and tools necessary to protect and support themselves during and after extreme weather events. Television, Internet, radio, and newspaper alerts can be used to inform the population about heat waves and other impending events, explain the dangers, identify at-risk groups, and discuss prevention or response measures (e.g., emergency hotlines) (Caruso and Posey 1985; U.S. EPA 2006).

*Community capabilities.* Adaptations that strengthen communities’ response to climate stressors can be accomplished in a number of ways. Some impacts can be ameliorated through physical design changes in neighborhoods or through early warning systems (U.S. EPA 2006). Policy makers and planners should focus on tailoring specific local responses to optimize the use of available community resources (Cutter and Emrich 2006). For instance, to reduce heat-related illness and mortality, communities can develop early warning and response or other surveillance systems (Bargagli et al. 2009; Bascetta 2006; Ebi et al. 2004). Community nurses and home health workers could reduce the risk of heat illness by educating patients about their vulnerability to heat (Fernandez et al. 2002). Local government agencies could distribute air conditioners or subsidize air conditioner use among vulnerable populations, although the distribution of electric fans is contraindicated, because the circulation of warm air in enclosed indoor spaces may actually increase heat stress (U.S. EPA 2006). Persons with special medical needs could be encouraged to register with their local emergency management agency to ensure that they will receive necessary services or evacuation assistance (Bascetta 2006). The effectiveness of communications can be strengthened by applying knowledge gained from similar efforts in other communities and by coordinating service providers. The principles of communication, effective emergency response planning and coordination, and targeted services are common themes across all effective adaptations to climate-related stressors.

*Tools and measures to improve adaptation and lessen vulnerability.* To understand and characterize vulnerability, Cutter et al. (2003) developed a Social Vulnerability Index that assesses the level of resilience to hazard events for U.S. communities, using county-level socioeconomic and demographic data. Similarly, Wilson et al. (2010) and Vescovi et al. (2005) proposed map-based climate change vulnerability indices. Vulnerability mapping can help planners, emergency managers, and first responders locate vulnerable individuals. To further aid prioritization and response, authorities can use rapid assessment tools to identify who needs help, how urgently, and the best course of action (Burnett et al. 2007). For example, to reduce the urban heat island effect, planners and developers could implement preemptive strategies (e.g., planting trees and other vegetation, installing green roofs, and utilizing light-colored pavements) (Ebi and Meehl 2007; Wilhelmi and Hayden 2010). These measures may provide multiple benefits, because reducing urban temperatures will reduce energy costs and improve air quality.

A wide range of tools and resources exist to help public health and emergency management officials to protect older Americans from hazards related to climate change. Educational materials and disaster checklists can be developed for distribution to older Americans, their families, and caregivers through social networks, community-based services, faith-based organizations, and health care providers (Eisenman et al. 2007).

## Discussion

There is strong evidence that older Americans will be more vulnerable than the general population to climate stressors. In light of the projected increase in the population of older Americans and the fact that much of that population is concentrated in regions likely to experience significant impacts from climate change, research to identify vulnerabilities and develop strategies to improve the resilience of older adults and their communities should be a priority. Key findings of this review include the following:

*Demographics.* Older Americans are a diverse and rapidly growing population. The number of older adults in the United States is expected to nearly double over the next 25 years. The physiological and social characteristics of older adults vary widely by age group, living situation, income, and other demographic features. Location matters—many older Americans live in regions that could be hard hit by extreme events associated with climate change.

*Primary climate stressors*. In the absence of effective adaptation, climate change is likely to disproportionately affect older adults. Projected climate stressors to which older adults are particularly sensitive due to biologic traits include extreme heat and other extreme weather events, degraded air quality, and increases in the risk of infectious diseases. Some of these impacts are projected to occur in places where older adults are heavily concentrated and likely to be most exposed. This confluence of sensitivity, exposure, and adaptive capacity determines older adults’ overall vulnerability to climate change.

*Factors affecting exposure*. Exposure is determined by local and individual factors. The quality and adequacy of buildings and infrastructure, as well as the availability of social services and community support networks, affect the degree to which older adults are likely to be exposed.

*Determinants of adaptive capacity*. Socioeconomic status and community resources strongly influence adaptive capacity and resilience. Financial resources allow communities to invest in effective technologies and infrastructure, provide social services, build strong institutions, and support vibrant and safe neighborhoods.

*Measures to reduce vulnerability*. Building capacity at both the community and individual levels is essential. Developing early warning, response, and surveillance systems, coupled with planning and development to enhance community resilience, can reduce the vulnerability of older adults and help them avoid or mitigate the impacts of climate stressors.

*Research gaps*. A rich store of demographic data is available on older Americans. The vulnerability of older adults to two climate stressors—​extreme heat events and hurricanes—has been widely reported. Less information is available on the vulnerability of older adults to other health-related climate impacts, such as river flooding, coastal flooding from sea level rise, droughts, wildfires, changes in air quality, and contaminated food and water supplies. More research is needed to identify and develop strategies for reducing the vulnerability of older adults to climate change, and for helping them prepare for and respond to emerging threats. Assessments of health impacts from climate change have not always included age-specific analyses. Improved consistency in this regard would draw attention to vulnerable subpopulations.

In addition, it would be useful to develop indicators (at local and regional scales) of older Americans’ overall vulnerability to the effects of climate change. Such indicators might include income, sex, rural or urban residence, owner-occupied home, infrastructure, education, population change, family structure/living alone, available health and social services, and special-needs populations (Cutter et al. 2003). These indicators could help to identify locations and populations that could be targeted for capacity-building and communication strategies to reduce vulnerability. Indicators can also be used to document changes across place and time, monitor vulnerable areas, and evaluate the effectiveness of adaptation strategies.

## Conclusions

Climate change will increase the exposure of a growing population of older Americans to a range of climate-related hazards. This increased exposure is of concern because older Americans are more sensitive than the general population to certain health impacts and generally have less capacity to adapt to climate stressors. The combination of these factors—exposure, sensitivity, and adaptive capacity—determines vulnerability.

More work is needed to understand the risks posed by climate change on older adults, and to communicate those risks to decision makers, public health and safety officials, and caregivers and advocates of aging populations. Public agencies can partner with nongovernmental organizations that work with older Americans to leverage communication efforts. More work is needed to develop adaptation strategies that reduce the vulnerability of older Americans to climate stressors. Given the projected growth of the older adult population, the U.S. health care system and other community services will be challenged to meet the growing demands by older adults caused by more frequent and more intense climate-related weather extremes. In the near term, it may be possible to build on and adapt some of the response strategies and communication approaches developed for heat waves and hurricanes to the broader set of climate change impacts.
